# Michigan University Medical Journal

**Published:** 1870-04

**Authors:** 


### Michigan University Medical Journal.

This journal is to be published under the auspices of the University of Michigan, the Faculty of the Medical Department acting as editors. Under this able management we confidently expect success in the undertaking. We welcome it right cordially to our list of exchanges.

